# Evaluating the importation of yellow fever cases into China in 2016 and strategies used to prevent and control the spread of the disease

**DOI:** 10.5365/wpsar.2018.9.1.007

**Published:** 2020-06-30

**Authors:** Chao Li, Dan Li, Shirley JoAnn Smart, Lei Zhou, Peng Yang, Jianming Ou, Yi He, Ruiqi Ren, Tao Ma, Nijuan Xiang, Haitian Sui, Yali Wang, Jian Zhao, Chaonan Wang, Yeping Wang, Daxin Ni, Isaac Chun-Hai Fung, Dexin Li, Yangmu Huang, Qun Li

**Affiliations:** a Public Health Emergency Center, Chinese Center for Disease Control and Prevention, Beijing, China.; b Department of Biostatistics, Epidemiology and Environmental Health Sciences, Jiann-Ping Hsu College of Public Health, Georgia Southern University, Statesboro, GA, United States of America.; c Department of Epidemiology and Biostatistics, School of Public Health, Peking University Health Science Center, Beijing, China.; d Beijing Center for Disease Control and Prevention, Beijing, China.; e Fujian Center for Disease Control and Prevention, Fuzhou, China.; f Shanghai Center for Disease Control and Prevention, Shanghai, China.; g Nanjing Center for Disease Control and Prevention, Nanjing, China.; h Institute for Viral Disease, Chinese Center for Disease Control and Prevention, Beijing, China.; ^i Public Health School, Peking University, Beijing, China.^.

## Abstract

During the yellow fever epidemic in Angola in 2016, cases of yellow fever were reported in China for the first time. The 11 cases, all Chinese nationals returning from Angola, were identified in March and April 2016, one to two weeks after the peak of the Angolan epidemic. One patient died; the other 10 cases recovered after treatment. This paper reviews the epidemiological characteristics of the 11 yellow fever cases imported into China. It examines case detection and disease control and surveillance, and presents recommendations for further action to prevent additional importation of yellow fever into China.

The 2016 yellow fever outbreak in Angola led to renewed attention to this often-fatal disease. Of the 4306 suspected cases reported, 376 individuals died (mortality rate, 8.7%). (*1*) The outbreak was declared to have been one of the largest and most challenging yellow fever outbreaks in recent years by the World Health Organization (WHO), in part because of its international spread to other countries, including China.

Yellow fever is a zoonotic disease that is endemic in tropical regions of Africa and South America. It is caused by the yellow fever virus, an arbovirus that belongs to the *Flavivirus* genus. (*2*) The virus is transmitted between humans, or from monkeys to humans, through the bite of infected mosquitoes belonging to the *Aedes* and *Haemogogus* genera, respectively. Yellow fever causes an estimated 30 000 deaths each year, most of which are in Africa, where more than 500 million people are at risk for yellow fever. (*3*) An additional 400 million people in Central and South America are also at risk. (*3*) Though *Aedes aegypti* mosquitoes are found in China (primarily in Fujian Province) and other parts of Asia, yellow fever had never, before 2016, been reported in China or any other part of Asia.

In 2015, more than 200 000 Chinese nationals were working or conducting business in Angola. (*4*) According to the General Administration of Quality Supervision, Inspection and Quarantine (AQSIQ), dozens of the Chinese nationals in Angola contracted yellow fever during the 2016 outbreak, resulting in eight deaths in Angola. (*5*) Facing the possibility of an imported yellow fever epidemic, China developed a national yellow fever control and prevention protocol, (*6*) and took steps to strengthen surveillance at airports and health-care facilities and to implement emergency vector surveillance. Despite these efforts, 11 Chinese nationals who were infected during the outbreak in Angola imported yellow fever into China in 2016.

Considering frequent travel, labour relationships, and close trade with endemic countries in South America and Africa, China faces a continued risk of yellow fever importation. Therefore, the purpose of this paper is to examine China’s response to the importation of its first yellow fever cases, particularly the emergency response, as well as case detection and disease surveillance and control.

## Methods

We obtained and analysed data collected as part of China’s emergency response. The following paragraphs describe the steps taken to identify and document the imported cases of yellow fever and to collect demographic and epidemiological data, as well as clinical information.

### Case definition

Suspected cases of yellow fever were identified by clinicians based on clinical manifestations consistent with yellow fever, which included fever, jaundice, liver and kidney dysfunction, vomiting, and bleeding, as well as epidemiological history (i.e. history of travel or residence in the last 14 days before symptom onset). Confirmed cases were defined as suspected cases that tested positive for yellow fever virus using nucleic acid testing. (*6*)

### Case discovery

Yellow fever cases imported into China were either discovered by AQSIQ staff during point-of-entry screening or later reported by treating health-care providers. Febrile passengers passing through the point of entry were identified via temperature screening and were transported to the hospital for diagnosis and treatment. Travellers with mild or moderate symptoms that had not been detected by entry screening were identified by health-care providers in hospitals while seeking medical care. Clinicians identified and documented symptoms consistent with yellow fever to identify suspected cases. When a suspected case of yellow fever needed to be confirmed, patient blood samples were sent to the provincial Center for Disease Control and Prevention (CDC) for laboratory testing by real-time reverse transcriptase polymerase chain reaction (rRT–PCR). (*7*) All information was reported to the local health administrative departments after disease confirmation. (*8*)

### Data collection

Once a suspected case was diagnosed by a laboratory, the provincial CDC conducted a field investigation to collect demographic and epidemiological information (including travel history, i.e. the dates of arrival in Angola and return to China and yellow fever vaccination status) and clinical information (including symptoms, time of symptom onset and date of hospital visit).

### Vector surveillance

Routine surveillance of *Aedes* density has been conducted in China for many years. According to the surveillance protocol, (*9*) all provinces are classified into three groups (high, middle and low) depending on the level of risk of mosquito-borne disease transmission. In high-risk areas, surveillance is conducted throughout the year. In middle- and low-risk areas, surveillance is conducted from May to November and from June to September. After each of the imported cases of yellow fever was identified, emergency monitoring was conducted to measure the mosquito density within a radius of 200 m of the patient’s residence. The Breteau Index (BI) was calculated to determine the number of positive containers per 100 households inspected. (*9*)

### Ethics approval and consent to participate

The case information was collected according to the regulations of the Law of the People’s Republic of China on the Prevention and Treatment of Infectious Diseases as a part of the emergency response, which was exempted from ethics approval and consent to participate.

## Results

### Case characteristics

A total of 11 cases of yellow fever were reported in China, all imported from Angola. All were Chinese nationals living in Luanda, the capital of Angola, at the time of the outbreak. Seven cases were residents of Fujian Province, two of Jiangsu Province, and one each of Zhejiang Province and Sichuan Province. All cases were identified in weeks 11 to 15 of 2016, approximately one to two weeks after the peak of the Angolan epidemic. The patient age range was 18–52 years (median: 42 years). Eight were male, and three were female. Eight were retailers, and three were labourers. Ten reported having been bitten by a mosquito at least six days before symptom onset; the other patient (case no. 2) was unsure if he had been bitten. Six patients reported having received yellow fever vaccinations. Case nos. 6, 7, 9 and 10 were vaccinated less than 14 days before system onset. Case no. 8 was vaccinated in China five years before symptom onset, and Case no. 11 was vaccinated in Namibia 10 months before the onset of illness (Table 1). (*10*)

**Table 1:** Demographic characteristics of imported cases of yellow fever

Case no.	Sex	Age (years)	Date of symptom onset	Reporting region	Location at time of diagnosis	Vaccination status	Reporting institution	Notes	Data source
**1**	**M**	**32**	**8 March 2016**	**Beijing**	**Beijing**	**Not vaccinated before symptom onset**	**AQSIQ**	**Died of the disease**	**10,19–21**
**2**	**M**	**46**	**5 March 2016**	**Shanghai**	**Shanghai**	**Unknown**	**AQSIQ**	**-**	**22**
**3**	**M**	**44**	**9 March 2016**	**Beijing**	**Beijing**	**Not vaccinated before travel to Angola**	**AQSIQ**	**-**	**10,23**
**4**	**M**	**44**	**11 March 2016**	**Beijing**	**Beijing**	**Not vaccinated before travel to Angola**	**AQSIQ**	**-**	**10,23**
**5**	**M**	**50**	**6 March 2016**	**Beijing**	**Beijing**	**Not vaccinated before travel to Angola**	**AQSIQ**	**Received treatments in multiple hospitals in Angola from 6 to 16 March 2016**	**10,24**
**6**	**F**	**42**	**11 March 2016**	**Fujian**	**Fuzhou**	**Not vaccinated before travel to Angola. Vaccinated in Angola on 7 March 2016, 3 days before symptom onset.**	**Medical Institution**	**-**	**10,25**
**7**	**M**	**42**	**17 March 2016**	**Fujian**	**Fuzhou**	**Vaccinated 7 days before symptom onset**	**Medical Institution**	**-**	**10,26**
**8**	**F**	**36**	**15 March 2016**	**Fujian**	**Fuzhou**	**Vaccinated 5 years before symptom onset**	**Medical Institution**	**-**	**10,26**
**9**	**F**	**53**	**13 March 2016**	**Fujian**	**Fuzhou**	**Vaccinated 1 day before symptom onset**	**Medical Institution**	**-**	**10,26**
**10**	**M**	**18**	**12 March 2016**	**Fujian**	**Fuzhou**	**Vaccinated 10 days before symptom onset**	**Medical Institution**	**-**	**10,27**
**11**	**M**	**29**	**5 April 2016**	**Beijing**	**Beijing**	**Vaccinated 10 months before symptom onset**	**AQSIQ**	**Sought medical care at a local hospital in Angola**	**10,11**

AQSIQ, General Administration of Quality Supervision, Inspection and Quarantine.

### Case detection

Ten cases received medical treatment in Luanda but were not diagnosed with yellow fever; the other case, having only mild symptoms, did not seek medical treatment before returning to China. Seven cases returned to China through the Beijing Capital International Airport; the other four entered through Shanghai. Six cases were reported within the city of entry, and five were eventually reported in Fujian Province, where they had sought medical care. Of the six cases discovered by AQSIQ, two cases with mild illness self-declared their symptoms at the time of entry (**Table 1**).

### Disease control and surveillance

The Chinese Government took steps to strengthen surveillance at airports and health-care facilities and implemented emergency vector surveillance in an attempt to prevent further cases of yellow fever. Specifically, the Government intensified multisectoral coordination and collaboration; strengthened surveillance, vector monitoring and risk assessment; enhanced clinical management of yellow fever cases; conducted vector control activities; carried out public risk communication activities; and deployed a medical team to Angola to provide yellow fever vaccination to unvaccinated Chinese nationals. (*11*)

## Discussion

We describe the 11 cases of imported yellow fever in China, most of which were discovered within two weeks after the peak of the outbreak in Angola in 2016. After the outbreak in Angola was announced, China quickly released a protocol for yellow fever prevention and control. (*6*) At the same time, AQSIQ, in the hope of preventing yellow fever from entering China, issued an announcement that included instructions for screening travellers and checking vaccination certificates. (*12*)

Several strategies were implemented to control the spread of yellow fever in China. First, all travellers from Angola were required to present yellow fever vaccination certificates. Those without a certificate were isolated at the point of entry or their place of residence for six days. Second, all travellers from affected countries were screened upon entry. Anyone who self-declared or who was suspected of having yellow fever was isolated at the entry point. There, AQSIQ staff administered an epidemiological survey and collected blood samples for testing. Third, travellers from Angola and other epidemic countries were required to perform self-health monitoring for six days after entering China. If suspicious symptoms occurred, the affected traveller was asked to report to a health-care provider, disclose their travel history and receive prompt treatment. Additionally, aircraft, containers and other cargo from the epidemic countries were targeted for mosquito control. It was also recommended that persons travelling to Angola and other epidemic countries should be vaccinated again for yellow fever before departure from China. For the imported yellow fever cases, emergent monitoring of mosquito-borne vectors was also performed.

Areas recommended for improvement include epidemic information sharing, risk warning and health education for Chinese nationals in Angola and other yellow fever-endemic countries. According to our investigation, Chinese nationals in Angola are generally employees sent by private companies or individual business people, primarily from Fuqing City in Fujian Province. More than 200 000 Chinese nationals live and work outside China, (*3*) so timely health-related communication between health officials, companies and overseas workers could help protect China’s expatriate population from public health threats in their countries of temporary residence. Overseas workers in Angola should have received a yellow fever vaccination before their departure from China. Information about the yellow fever epidemic, if received from their companies or Chinese health officials, might have encouraged personal prevention measures such as mosquito-avoidance precautions. Information on travellers with yellow fever, especially those who returned to China for treatment, should have been reported by the employing companies to the Chinese embassy or Government, which would have provided valuable information for disease prevention. Required vaccination and improved communication are also crucial for individual business people who are travelling to yellow fever-endemic countries. All inbound passengers should be required to present proof of vaccination if they are arriving from yellow fever-endemic countries. Health education materials (e.g. videos, posters, warning signs, brochures and text messages) could be provided at the points of entry by inspection and quarantine officials to encourage inbound passengers to self-declare symptoms of a potential communicable disease. (*13*)

Additional strategies have been identified and are recommended for reducing the risk of importation and spread of infectious diseases in China. For instance, China could strengthen regulations and legislation to put an end to the fabrication of false yellow fever vaccination certificates, a practice used to circumvent the vaccination regulations of the International Health Regulations, or IHR (2005). Globally, governments, including that of China, could ensure their citizens receive yellow fever vaccination when travelling to countries that recommend it, could tighten border controls to ensure incoming visitors from yellow fever-endemic countries have proof of vaccination and could make public policies a priority in the prevention of diseases among travellers. (*14*)

The active period for the *Aedes aegypti* mosquito in Fujian Province was reported to be from May to October in 2016. (*15*) However, the result of emergency monitoring (BI: 15) indicated continued transmission risk of mosquito-borne diseases after the peak period. Thus, the public should be educated to eliminate containers that can hold water in which mosquitoes may breed.

### Risk of disease importation

Due to frequent travel and close trade with yellow fever-endemic countries in Africa and South America, China faces a continued risk of yellow fever importation as travel volume has increased. Travel patterns to and from yellow fever-endemic regions in relation to China indicate that Angola sends the second-highest number of travellers into China and also receives the second-highest number of Chinese visitors. (*14*) During the years 2010–2030, tourist arrivals in Asia and the Pacific are expected to increase by 331 million, bringing the total number of tourists to about 535 million in 2030. With this increase in travel, there will be a concomitant increase in the importation of infectious diseases. Due to the presence of the urban mosquito vector, *Aedes aegypti*, among large unvaccinated populations, 1.8 billion people in Asia were put at risk for yellow fever by international travellers during the 2016 outbreak. (*14*) In Angola, by September 2016, near the end of the 2016 yellow fever outbreak, 884 laboratory-confirmed cases of yellow fever had been reported, with 373 deaths. The confirmed cases of yellow fever in China were the first-ever cases to be imported into Asia. (*14*)

In 2012, a total of 475 761 air passengers travelled to China from yellow fever-endemic countries. Of those, 195 291 travelled from the South American countries of Argentina, Brazil, Columbia and Venezuela, and 104 854 travelled from the African countries of Angola, Ethiopia, Ghana and Nigeria. During that same year, 466 832 air passengers from China travelled to yellow fever-endemic countries. The importation of yellow fever from endemic countries by unvaccinated Chinese workers is a serious concern, as they are apparently able to circumvent the mandated IHR (2005) regulations that require proof of vaccination for entry into China from certain yellow fever-endemic countries, including Angola. (*14*)

### Vaccinations

Finally, although yellow fever is a vaccine-preventable disease, the vaccination rate of yellow fever in Chinese nationals in Angola is estimated to be very low. (*16*) Vaccinations should be required for all Chinese nationals going to or returning from countries where yellow fever is endemic, as per the WHO recommendations. (*11*) This would protect Chinese citizens who are residing in countries such as Angola and Brazil, where the risk of contracting yellow fever is substantial.

The WHO risk assessment report of yellow fever infection in non-immunized travellers underlined the need to reinforce the implementation of yellow fever vaccination requirements and highlighted the risk of international spread of the disease through non-immunized travellers. (*11*) A safe and effective vaccine for yellow fever has been available for more than 50 years. The licensed, live attenuated yellow fever vaccine produces immunization within 10 days and has a long duration of immunity. However, it is in short supply, with only about 80 million doses produced annually. An estimated half a million doses of the vaccine would be needed annually to cover the Chinese population travelling to yellow fever-endemic countries. Yellow fever 17D vaccine is manufactured in China for the domestic market and therefore is available, although the supply is limited. (*12*)

Routine vaccination for children living in countries at risk for yellow fever is also recommended by WHO. (*3*) The vaccine confers long-term protection (10 years or more, possibly lifelong) within 10 days for more than 90–95% of individuals who receive the vaccine. Within 30 days after vaccination, 99% of those immunized develop immunity. A single dose is likely to provide lifelong immunity. The yellow fever vaccination certificate is now valid for the duration of the life of the person vaccinated. (*17*)

Among the 11 cases, there were two cases with vaccination failure. Although vaccination failure for yellow fever is unusual, some studies showed up to 26% seronegativity in vaccines after mass immunization campaigns. (*18*) External factors such as improper cold chain handling, storage and administration may be the cause of failure.

In conclusion, we have described the first-ever importation of yellow fever cases in China, discussed the methods used for case detection and prevention of imported infectious disease, and provided several recommendations for disease prevention and control. Experiences gained from the response to imported yellow fever cases in 2016 can be used to protect Chinese travellers from yellow fever and to prevent new importations of the disease.
